# Variant angina in moyamoya disease – a correlative etiology and different presentation: a case report

**DOI:** 10.1186/s13256-015-0537-4

**Published:** 2015-04-22

**Authors:** Woong Choi, Yu Na Kim, Kyung-Hee Kim

**Affiliations:** Division of Cardiology, Department of Internal Medicine, Sejong General Hospital & Sejong Heart Institute, Hyohyunro 489 street, Bucheon, Kyunggi-do Republic of Korea

**Keywords:** Coronary artery disease, Moyamoya disease, Variant angina

## Abstract

**Introduction:**

Moyamoya disease is characterized by progressive steno-occlusive changes of the distal internal carotid and developed collateral vasculature, so called ‘moyamoya’ vessels at the base of the brain. Variant angina is a rare occurrence in patients with moyamoya disease.

**Case presentation:**

Here we report the case of a 41-year-old Korean woman who developed chest pain after indirect revascularization surgery of moyamoya disease. A treadmill test and an exercise stress echocardiograph showed positive results, but there was no significant major coronary arteries stenosis. Suspicious of vasospasm, we conducted an ergonovine spasm stimulation test, which demonstrated tight stenosis of her proximal left anterior descending artery. At the site of spasm, intravascular ultrasound virtual histology showed intraluminal fibrous plaque.

**Conclusion:**

Physicians who follow up patients with moyamoya disease would need to be aware of the possibility of cardiac ischemia as well as neurological manifestations.

## Introduction

Moyamoya disease is an uncommon condition characterized by spontaneous bilateral stenosis or occlusion of the terminal portion of the internal carotid artery and other proximal portions of cerebral arteries accompanied by collateral branches which on angiography seem hazy like a puff of cigarette smoke [1]. Although the etiology of moyamoya disease is unknown, reduced vasoreactivity of cerebral vessels has been reported in moyamoya disease [2]. Autopsies have revealed decreased vessel size with fibrous thickening of the intima with minimum lipid deposition, internal elastic lamina degeneration and no substantial inflammatory cell infiltration is seen in the vascular wall [3]. There have been several reports of involvement of extracranial vessels, especially the renal arteries, in moyamoya disease [4] but few studies have provided evidence as to whether these patients developed ischemic heart disease [5,6]. Here we report the first case of variant angina pectoris proven by ergonovine provocation test associated with moyamoya disease.

## Case presentation

**Figure 1:**
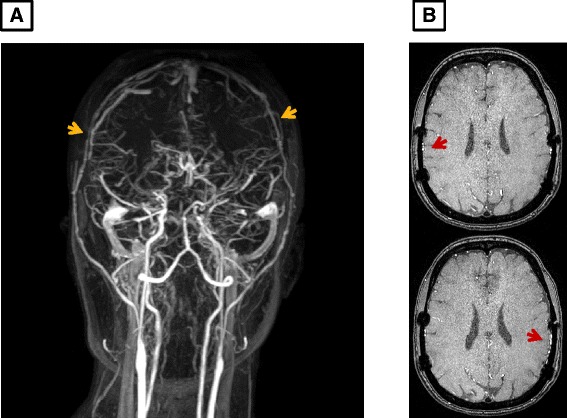
Brain magnetic resonance imaging and angiography after indirect revascularization (arrows). **A**: Magnetic resonance angiography, coronal view; **B**: axial view.

A 41-year-old Korean woman was admitted to our hospital with the complaint of recurrent chest pain in February 2014. The pain felt like squeezing of her chest, which radiated to her left arm, occurred usually in the morning or when it was getting cold, was aggravated by stress and relieved by sublingual nitroglycerin spray. She was diagnosed with moyamoya disease 8 years ago in 2006 and received indirect revascularization, encephalo-duro-arterio-synangiosis (Figure 1A and B), she has had chest pain since then. One month later, she underwent a cardiology work up including exercise stress echocardiography and coronary angiography (CAG) at another hospital. Exercise stress echocardiography showed left anterior descending artery (LAD) territory ischemia but the CAG showed no significant coronary artery stenosis. She was diagnosed with syndrome X and then referred to a psychiatrist at that time. She had never smoked cigarettes, she was not a social drinker and she denied a family history of cardiovascular disease. She has complained of intermittent chest pain and the intensity of it increased before she visited our hospital.

**Figure 2:**
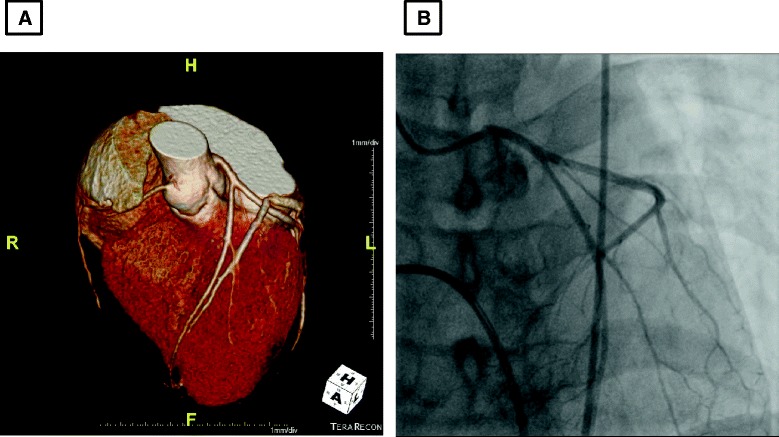
Coronary artery image demonstrating a hypoplastic right coronary artery without significant luminal narrowing of major vessels. **A**: Coronary computed tomography angiography; **B**: coronary angiography showed no coronary artery lesion.

**Figure 3:**
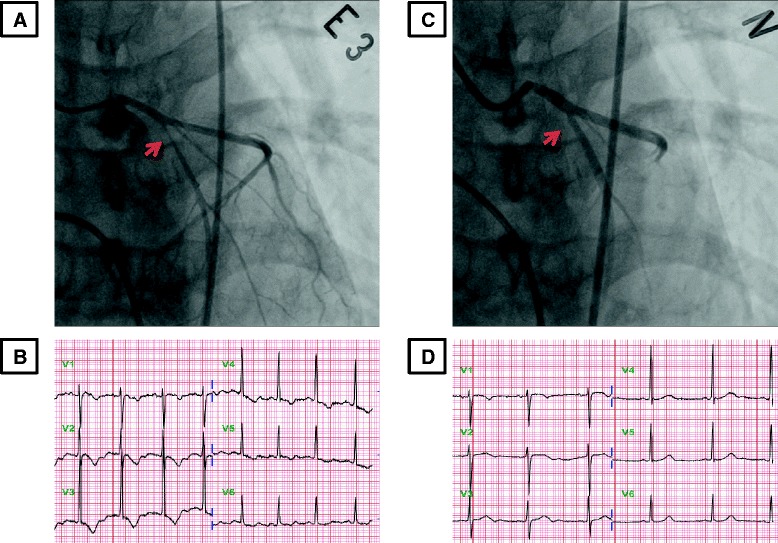
Coronary angiography. **A**: Coronary angiography demonstrated proximal left anterior descending artery focal 95% stenosis (arrow in A) after twice intravenous ergonovine administration. **B**: The patient complained of typical chest pain and there was ST-segment changes after ergonovine administration. **C**: After injection of intracoronary nitroglycerin, the stenosis was completely resolved (arrow in C). **D**: The T wave was normalized after injection of nitroglycerin.

**Figure 4:**
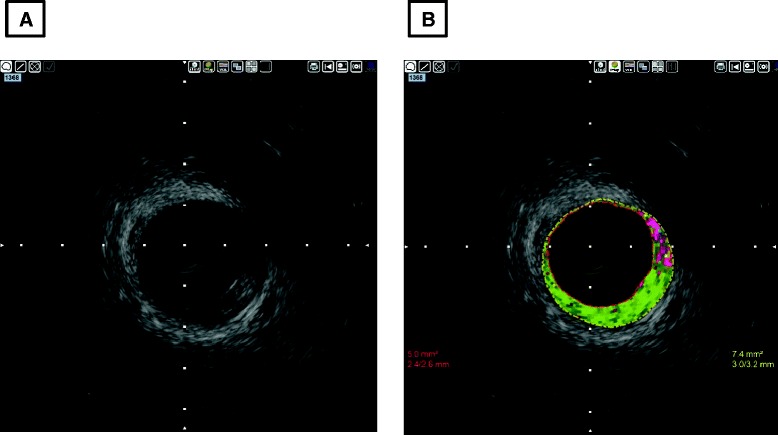
Intravascular ultrasound virtual histology image for the site of coronary spasm. **A**: The coronary lumen has 33% plaque. **B**: The component of plaque is mostly fibrous material.

The results of laboratory tests including creatine kinase, creatine kinase-MB, and troponin T were within normal levels and electrocardiography showed sinus rhythm with no ST-segment abnormality. Echocardiography showed normal LV function and no regional wall motion abnormality. A treadmill test showed positive result of ST-segment depression and chest discomfort at stage 4. Coronary computed tomography angiography and CAG (Figure 2A and B), showed a hypoplastic right coronary artery without significant coronary artery stenosis. We performed a coronary spasm provocation test, suspicious of vasospastic angina. Ergonovine 0.2mg was injected three times intravenously at 5-minute intervals. We achieved cine angiogram at 90 seconds after each ergonovine injection. Coronary angiography demonstrated proximal LAD focal 95% stenosis after two administrations of intravenous ergonovine; the patient complained of typical chest pain and there were ST-segment changes (Figure 3A and B). After injection of intracoronary nitroglycerin, the stenosis was completely resolved and her T wave was normalized (Figure 3C and D). An intravascular ultrasound virtual histology (IVUS-VH) image for the site of the spasm showed fibrous plaque occupying 33% of the coronary luminal area (Figure 4).

Finally the patient was diagnosed with variant angina and we prescribed calcium channel blocker, nitrate and moderate dose of statin. Her chest pain seemed to be controlled with those medications; however, her chest pain recurred several months later. We added nicorandil and non-dihydropyridine calcium channel blocker; we are monitoring her every month at our out-patient clinic and up to now there have been no further events.

## Discussion

The incidence of moyamoya disease is higher in East Asia, such as Japan and Korea, than in Western countries. Moyamoya disease is generally manifested by ischemic or hemorrhagic stroke (that is, hemiplegia, headache, numbness, and so on) and occasionally accompanied by extracerebral disease (autoimmune disease, connective tissue disease and extracranial arterial system) [7]. Although an association of moyamoya and renovascular disease is well described [4], reports of coronary artery disease in moyamoya disease are rare. To the best of our knowledge only four cases of moyamoya disease with suspicious variant angina have been reported, but no studies have provided evidence as to variant angina proven vasospasm and shown virtual histology of coronary artery as in this patient [6,8].

Spontaneous occlusion of cerebrovascular moyamoya disease is characterized by occlusive or stenotic lesions at or around the terminal portions of the internal carotid arteries and abnormal vascular networks at the base of the brain. The obstructive lesions are caused by fibrous thickening of the intima with minimum lipid deposition, the internal elastic lamina is well preserved, and no substantial inflammatory cell infiltration is seen in the vascular wall.

Many reports showed that the pulmonary, renal and pancreatic arteries in patients with moyamoya disease may manifest histopathologic changes similar to the stenotic changes of the intracranial internal carotid arteries [9,10]. Even though a few cases of coronary artery stenosis in patients with moyamoya disease have been reported, the histopathology of these coronary lesions is similar to the findings of the carotid arteries of patients with moyamoya. Pathology has shown soft intimal proliferation with minimum lipid deposition which significantly differs from typical atherosclerotic plaques. Usual coronary artery spasm very often occurs at the site of atheromatous plaques, which suggests that atheromatous plaques could magnify the lumen diameter reduction. In this case we showed coronary artery spasm within the site of fibrous plaque in a patient with moyamoya disease. An IVUS-VH finding of CAD in moyamoya disease reported by Lee *et al*. showed intracoronary fibrous plaque similar to our case [11]. The cellular proliferation and vascular dysregulation in moyamoya and vascular smooth muscle cell hyperreactivity in variant angina were suggested to have a common pathogenic link. This finding indicates that moyamoya disease may actually be an intracranial manifestation in a systemic arterial disorder.

Another possible link between these disorders is genetic and ethnic factors. The pathogenesis of moyamoya disease is still unclear. Many reports suggest that genetic factors might also contribute to the development of the disease; data from an epidemiological study showed a high incidence among the Japanese and Asian population, together with a familial occurrence in approximately 10 to 15% of cases, which strongly suggests a genetic etiology. An analysis of an American survey of 298 patients with moyamoya disease found an annual prevalence of 0.086 per 100,000 which was significantly lower than the 0.35 reported in a nationwide Japanese survey [12,13]. Similarly, an analysis of the epidemiological features of this disease in Hawaii, where a high proportion of the population are Asian and Pacific Islanders (56%), found incidence and prevalence of moyamoya disease to be higher than in the rest of the USA because of the larger percentage of people of Asian ethnic origins [14]. The role of genetic factors in the pathogenesis of variant angina has also been suggested by the observation of a different prevalence of vasospastic angina in Japanese and White people. In the USA, the frequency is about 4% of patients who underwent coronary angiography with positive ergonovine test, whereas in Japan, positive study rates are in the rage of 30% [15,16]. Japanese patients are much more likely to develop coronary artery vasospasm than Caucasian patients.

Both Moyamoya disease and variant angina are more frequent in Asian populations, including Japanese, than in Caucasian populations. Although a specific associative mechanism between both diseases is not clear, a racial or a genetic characteristic might be related to the pathogenesis. Further epidemiologic and genetic data are needed to elucidate the correlated mechanism of these diseases.

From the clinical point of view, it is important to cure the ischemic attack of patients who have both moyamoya and variant angina. This case suggests that moyamoya disease might be regarded as a systemic vascular disorder, and evaluation of the extracerebral cardiovascular system is also required in this disease.

## Conclusions

The etiology of moyamoya disease is still unknown, but the disease can be regarded as a systemic arterial disease. Physicians who follow up patients with moyamoya disease need to be aware of the possibility of cardiac ischemia as well as neurological manifestations. We believe that educating patients about cardiac symptoms is also important so that these patients will seek medical attention if cardiac symptoms manifest.

## Consent

Written informed consent was obtained from the patient for publication of this case report and any accompanying images. A copy of the written consent is available for review by the Editor-in-Chief of this journal.

## Abbreviations

CAG: Coronary angiography
IVUS-VH: Intravascular ultrasound virtual histology
LAD: Left anterior descending artery
